# Acute Effects of Low-Intensity Blood-Flow-Restricted Walking on Pain Sensitivity, Joint Range of Motion, and Myofascial Stiffness in Healthy Adults

**DOI:** 10.3390/jcm15031052

**Published:** 2026-01-28

**Authors:** Robert Schleip, Juliane Herzer Santana, Christoph Egner, Andreas Brandl, Lea Overmann

**Affiliations:** 1 Department for Medical Professions, Diploma University of Applied Sciences, 37242 Bad Sooden-Allendorf, Germanychristoph.egner@diploma.de (C.E.); andreas-rudi.brandl@tum.de (A.B.); 2 Department of Conservative and Rehabilitative Orthopedics, TUM School of Medicine & Health, Technical University of Munich, 80809 Munich, Germany; 3 Triagon Academy, MRS 1331 Marsa, Malta; 4 Private Clinic Overmann, 59229 Ahlen, Germany; overmann.lea@gmx.de; 5 Fascia Research Project, 80799 Munich, Germany

**Keywords:** blood flow restriction, walking, pain modulation, range of motion, myofascial stiffness, exercise physiology

## Abstract

**Background/Objectives**: Blood Flow Restriction training has been suggested as a method to enhance strength and neuromuscular adaptations at low exercise intensities. Early reports indicate potential effects on pain perception, myofascial stiffness, and flexibility; however, the evidence remains inconsistent. **Method**: Twenty-two healthy adults participated in a randomized, within-participant, contralateral-controlled design, performing 5 min of treadmill walking (4–5 km/h) with and without blood flow restriction at 70% arterial occlusion pressure. Pressure pain threshold, hip range of motion, and hamstring stiffness were measured before and after the intervention. Adverse effects were recorded. **Results**: Changes in pain threshold, range of motion, and myofascial stiffness were similar between conditions. The pressure pain threshold decreased slightly in both conditions, regardless of BFR, while range of motion and stiffness remained unchanged. Mild, short-lasting sensations (cuff pressure, erythema, tingling) were reported, with no adverse events. **Conclusions**: A single short session of low-intensity BFR walking did not change pain sensitivity, flexibility, or myofascial stiffness in healthy adults. The protocol was well tolerated. Repeated or longer interventions may be needed to see measurable effects.

## 1. Introduction

Blood flow restriction training (BFRT) has become increasingly popular in clinical rehabilitation, sports medicine, and performance training because it can produce physiological changes with low mechanical loads. By applying external pressure to the limb’s proximal segments, BFRT partially restricts arterial inflow and significantly limits venous outflow, creating a hypoxic, metabolically demanding environment in the muscle even during low-load exercise [1,2]. This metabolic stress prompts afferent feedback, enhances the recruitment of type II muscle fibers, and boosts systemic hormonal and autonomic responses, which are linked to strength improvements, muscle growth, and pain relief [3,4]. As a result, BFRT is especially useful for populations unable to tolerate high mechanical stresses, such as postoperative patients, people with chronic pain, or those recovering from musculoskeletal injuries.

Beyond strengthening effects, an emerging body of evidence suggests that BFRT may acutely modulate pain perception. Several studies have demonstrated significant increases in pressure pain thresholds (PPT) following low-load BFRT, both locally and at distant sites, indicating the possible involvement of endogenous hypoalgesic mechanisms such as exercise-induced hypoalgesia and altered nociceptive signaling [5,6]. These findings have positioned BFRT as a potential adjunct in pain-focused rehabilitation strategies, where traditional exercise modalities may be insufficient to induce meaningful analgesic responses.

While most BFRT research has focused on resistance exercise, interest in using BFRT during aerobic activities—particularly walking—has grown. BFRT-walking offers a low-impact, accessible option, making it suitable for clinical settings or early-stage rehabilitation. Studies on BFRT-walking show improvements in functional performance, aerobic capacity, and muscle adaptations even at slow walking speeds and low intensity [7]. Despite its increasing popularity, evidence of immediate mechanistic effects—such as changes in pain sensitivity, joint mobility, or myofascial stiffness—remains limited and inconsistent. Some studies report higher PPTs after BFRT-walking, while others see little or no change, suggesting responses may depend on factors such as occlusion pressure, walking speed, intervention duration, or the participant’s physiological state.

Joint mobility and myofascial tissue properties are additional important factors that may be affected by BFRT. Repeated cycles of ischemia and reperfusion, metabolite buildup, and localized swelling could alter myofascial stiffness or range of motion (ROM), potentially influencing clinical decisions when prescribing BFRT. However, data on BFRT’s immediate effects on ROM and myofascial stiffness are limited. Most research focuses on long-term adaptations, leaving the initial responses during simple tasks like walking largely unexplored. Filling this gap is crucial for optimizing BFRT settings and practicing safely based on solid evidence.

Given the strong interest in BFRT walking and the need to better understand its immediate physiological and mechanical effects, further research is warranted. Existing studies mainly focus on long-term training outcomes, leaving short-term responses—especially those related to pain sensitivity, passive flexibility, and myofascial—understudied. Understanding these quick responses is crucial for assessing the therapeutic benefits and risks of BFRT walking in rehabilitation and sports medicine.

Therefore, the present study aimed to examine the immediate effects of low-load blood-flow-restricted walking on pressure pain thresholds, passive straight-leg raise (SLR) range of motion, and myofascial stiffness in healthy young adults.

## 2. Method

The study employed a randomized, within-participant, contralateral controlled design. Both experimental conditions—walking with BFR and walking without BFR—were applied simultaneously to opposite limbs during the same session. This approach allowed each participant to serve as their own control while minimizing inter-individual variability. Before data collection, the study protocol was reviewed and approved by the Ethics Committee of DIPLOMA University, with reference number 1137/2024, approved date: 30 August 2024. The study was conducted in accordance with the ethical principles outlined in the Declaration of Helsinki and was registered with the German Clinical Trials Register on 15 September 2025 (DRKS00037917). All participants received both written and oral information about the study procedures, potential risks, and expected benefits, and they provided written informed consent before participating.

### 2.1. Participants

An a priori power analysis (G*Power 3.1; mixed 2 × 2 repeated-measures ANOVA; primary test: condition × time interaction) assumed α = 0.05, 1 − β = 0.80, and a 5–10% dropout. Given that evidence on acute BFR(-walking) effects on pain sensitivity is limited and inconsistent, we chose a conservative small-to-medium interaction effect (f = 0.23, slightly below Cohen’s conventional medium effect of f = 0.25). This resulted in a required sample size of n = 22 [6]. Participants were recruited from the general population in Leipzig, Germany, via social media announcements. Interested individuals received an email with detailed study information and a screening questionnaire. The inclusion criteria included being between 18 and 65 years old and reporting good overall health. Exclusion criteria consisted of any prior experience with BFRT and the presence of contraindications for BFRT identified through a structured risk assessment.

**Figure 1:**
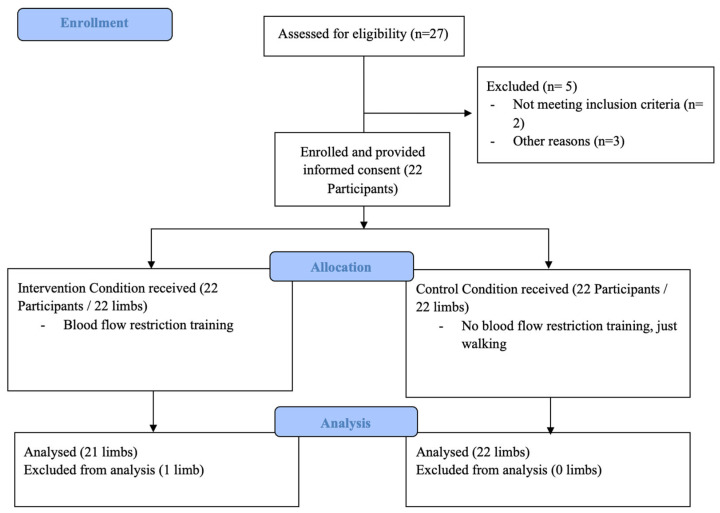
Study enrollment.

The study investigator developed the screening questionnaire based on previously published BFRT safety guidelines and risk assessment tools [8,9,10]. It evaluated anthropometric measurements, limb dominance, lifestyle habits, medical history, and symptoms. A points-based system adapted from Nakajima et al. (2011) [8] was used to classify BFRT-related risk; individuals surpassing a specific risk threshold were excluded. Of the 27 volunteers initially interested, two were excluded due to a high risk score, and three could not attend the measurement session due to acute illness. Twenty-two participants completed the study. Each participant provided measurements from two limbs (one limb assigned to the BFR condition and the contralateral limb serving as control). Due to a technical issue, unilateral outcome data were missing for one limb in one participant. Therefore, the final dataset comprised 43 analyzable limbs (10 women, 12 men). The CONSORT participant flow diagram is shown in Figure 1. Study enrollment.

### 2.2. Randomization and Blinding

Each participant’s dominant leg was identified through a questionnaire and used for randomization. The assignment of the BFRT condition to either the dominant or non-dominant leg was performed using block randomization (11 blocks with two allocation patterns each), generated with the ResearchRandomizer tool [11]. For each participant, one leg was designated as the BFRT intervention leg, and the other as the non-BFRT control. Due to the nature of the intervention, participants could not be blinded to the presence of the cuff. The study investigator conducted all measurements and was therefore not fully blinded to leg allocation; however, they did not know whether the cuffed leg was the dominant or non-dominant side—a second person assisted with protocol adherence and documentation.

### 2.3. Measurement Instruments

A series of validated measurement tools was used to evaluate pressure pain sensitivity, passive range of motion, and myofascial stiffness. The same trained investigator conducted all assessments to reduce inter-rater variability.

#### 2.3.1. indentoPro (indentoPro v.03.04)

The indentoPro was used to assess myofascial stiffness and PPT of the quadriceps and hamstring muscles. The device applies a controlled mechanical indentation, capturing force–displacement data to calculate stiffness in Newtons per millimeter (N/mm). For stiffness measurements, an indentation depth of 5 mm was used at a consistent loading rate. Each measurement was repeated three times within 15 s, and the device automatically calculated the mean value, provided the coefficient of variation was less than 10%. For the PPT, participants were instructed to indicate verbally when pressure became painful. Three trials were conducted at each site, and the average values were used for analysis. The indentoPro shows high reliability for stiffness measurement and has demonstrated validity comparable to that of established digital algometers for PPT measurement. The indentoPro is reliable and valid compared to other stiffness-measuring devices [12]. In addition, both intra- and inter-tester reliability have been demonstrated to be high [13].

#### 2.3.2. EasyAngle Digital Goniometer (Meloq^®^, Stockholm)

Passive hip flexion ROM was measured using the EasyAngle goniometer, which offers digital angle readings with high intra- and inter-rater reliability. The passive SLR test was conducted with the participant lying on their back and their knee fully extended. The device was aligned along the fibular shaft, between the fibular head and lateral malleolus, to record hip flexion angles. One measurement was taken for each leg at every time point [14,15]. The SLR was selected as a clinically relevant proxy for posterior-chain flexibility and hip flexion range of motion.

#### 2.3.3. Pulse Oximeter (Kernmed OLED A310)

A pulse oximeter was attached to the second toe of the intervention leg to measure the arterial occlusion pressure (AOP). The BFRT cuff was slowly inflated until the pulse waveform disappeared, indicating complete arterial occlusion. This approach provides a practical alternative to Doppler assessment in healthy individuals and is commonly employed in BFRT field studies [16,17].

### 2.4. Blood Flow Restriction Equipment

BFRT was conducted using conically shaped pneumatic cuffs designed for the lower limbs (Leg Cuffs V3.1, cuff width 10.5 cm, Fit Cuffs ApS, Aarhus, Denmark), connected to a manual pressure gauge. Arterial occlusion pressure (AOP) was measured while the client was in a supine position, with the cuff positioned as proximally as possible on the thigh of the limb to be treated. A pulse oximeter (Kernmed OLED Finger Pulse Oximeter A310, Kernmed, Troisdorf, Germany) was attached to the second toe of the same foot. The cuff was gradually inflated until the pulse oximetry signal disappeared, indicating complete arterial occlusion. This pressure was recorded as the individual’s AOP. An illustration of the cuff placement used during the BFRT walking protocol is provided in Supplementary Materials.

### 2.5. Intervention

After completing the baseline measurements, the BFR cuff was placed as proximally as possible on the intervention leg while the participant remained supine. The cuff was then inflated to the individually determined AOP. Following a brief reperfusion period, the cuff was re-inflated to 70% of AOP, which served as the training pressure. This pressure range was selected based on current BFRT recommendations, indicating that training pressures between 40% and 80% of AOP are generally effective for eliciting physiological responses [1,18]. Higher pressures may increase cardiovascular strain and the risk of soft tissue injury, especially when AOP is measured in positions associated with elevated occlusion pressures [2,19,20].

The exercise intervention involved a single session of low-intensity treadmill walking on a motorized treadmill (Desmo E, WOODWAY GmbH, Weil am Rhein, Germany). Participants walked at 4–5 km/h, simulating light, everyday physical activity. Before starting, they received detailed instructions and a demonstration on how to mount and dismount the treadmill safely. Once the walking speed stabilized, cuff pressure was re-checked to maintain approximately 70% of AOP. The walk lasted five minutes. Immediately after finishing, the cuff was fully released and removed, and all post-intervention measurements were taken promptly, following the same order as the baseline assessments.

The contralateral leg acted as the control condition and was exposed to the same five-minute treadmill walking session without BFR. Each participant thus provided paired data: one leg undergoing BFRT plus walking, and the other leg walking only.

### 2.6. Statistical Analysis

Statistical analyses were performed at the limb level within a within-participant design (BFR limb vs. contralateral control limb). For participants with unilateral missing data, the affected limb was excluded from the respective analyses; thus, analyses were conducted using all available limb data (total n = 43 limbs).

All statistical analyses were conducted using R, version 2025.05.1 (R Foundation for Statistical Computing, Vienna, Austria). Raw data were analyzed without any transformations. Means, standard deviations (SD), and 95% confidence intervals (CI) for all variables (PPT, ROM, tissue stiffness) were calculated for each condition (BFR vs. control) and each time point (pre vs. post). Since every participant completed both conditions in a contralateral controlled design, the effects of BFR were examined using a two-way mixed ANOVA (within-between-subjects design) with the between-subjects factor condition (BFR vs. control) and the within-subjects factor time (pre vs. post). The interaction term (condition × time) assessed whether changes over time differed between conditions. Significant pairwise comparisons were performed using Tukey’s HSD, with Cohen’s d as the effect size. All data met the criteria for parametric testing based on the Shapiro–Wilk normality test and Levene’s test for homogeneity of variances. The significance level was set at *p* < 0.05. Along with *p*-values, effect sizes (partial eta squared, ηp^2^) and mean differences with 95% confidence intervals (CIs) were calculated to indicate the effect sizes.

## 3. Results

A total of 22 participants completed the study. Due to unilateral missing data in one participant (technical issue), the final analyses included data from 43 limbs (21 BFR limbs and 22 control limbs). Baseline characteristics of the sample are shown in Table 1. The descriptive data of the outcomes are shown in Table 2.

### 3.1. Pressure Pain Threshold

**Figure 2:**
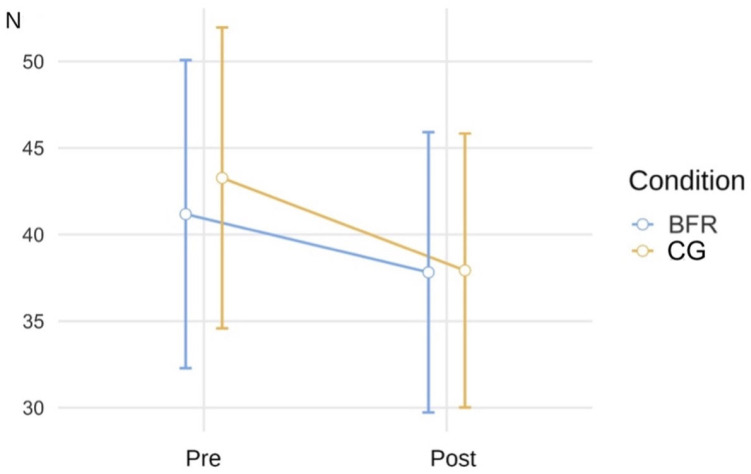
Mean pressure pain threshold values at pre- and post-measurement.

Pressure pain threshold decreased slightly from pre- to post-measurement in both conditions. The two-way mixed ANOVA revealed a significant main effect of time, indicating a general reduction in PPT following the walking intervention. However, no significant condition × time interaction was observed, suggesting that this change was not specific to the BFR condition. Post hoc analyses showed a statistically significant pre–post decrease in PPT only within the control condition, whereas the corresponding change in the BFR condition did not reach statistical significance (Table 3, Figure 2).

Figure 2 Mean pressure pain threshold (PPT) values at pre- and post-measurement for the BFR and control (CG) conditions. Error bars represent 95% confidence intervals. PPT slightly decreased over time in both conditions, with no condition × time interaction. N = Newton, BFR = Blood Flow Restriction

### 3.2. Range of Motion

The passive straight-leg raise range of motion remained largely unchanged from pre- to post-intervention in both conditions. In the BFR condition, ROM decreased slightly from 80.4 ± 16.1° at baseline to 78.2 ± 17.7° post-intervention, whereas the control condition showed virtually no change (77.9 ± 16.2° pre vs. 77.8 ± 16.4° post).

The two-way mixed ANOVA revealed no significant condition × time interaction (F(1,41) = 2.05, *p* = 0.159, ηp^2^ = 0.05), no main effect of condition (F(1,41) = 0.086, *p* = 0.771), and no main effect of time (F(1,41) = 2.24, *p* = 0.143).

### 3.3. Myofascial Tissue Stiffness

Myofascial stiffness showed only minimal variation across time and conditions. In the BFR condition, stiffness increased slightly from 0.90 ± 0.29 N/mm at baseline to 0.94 ± 0.26 N/mm post-intervention. Similarly, the control condition showed a small increase from 0.90 ± 0.28 N/mm to 0.96 ± 0.41 N/mm.

The two-way mixed ANOVA revealed no significant condition × time interaction (F(1,41) = 0.08, *p* = 0.778, ηp^2^ = 0.05), no main effect of condition (F(1,41) = 0.01, *p* = 0.931), and no main effect of time (F(1,41) = 1.96, *p* = 0.469).

### 3.4. Adverse Events

No serious adverse events occurred during the study. By the end of the intervention, 13 of 22 participants (59%) reported no adverse effects. Nine participants (41%) experienced mild, temporary sensations, such as muscle burning, pressure, or discomfort under the cuff, temporary redness, or tingling during reperfusion, primarily in the BFR condition. All symptoms resolved on their own without further treatment (Table 4).

## 4. Discussion

This study aimed to investigate the immediate effects of low-load blood flow-restricted walking on PPT, passive hip range of motion, and myofascial stiffness of the quadriceps and hamstrings in healthy young adults. Overall, the results indicate that a single five-minute treadmill walk, whether performed with or without blood flow restriction, did not cause significant changes in tissue properties or joint mobility. These findings help clarify the short-term physiological responses to BFRT walking and add to the limited evidence on acute neuromechanical adaptations to low-intensity BFRT protocols.

A key methodological consideration in BFRT research is accurately determining arterial occlusion pressure. Although Doppler ultrasound is regarded as the gold standard due to its high sensitivity and accuracy [1,2], it is not always practical for routine use because it requires specialized equipment, operator skill, and additional time. As a helpful alternative, pulse oximetry is frequently used in applied BFRT settings. It can provide fairly reliable AOP or LOP (limb occlusion pressure) estimates in young, healthy individuals when standardized protocols are followed [3,17]. However, its accuracy diminishes under conditions of low perfusion—an unavoidable consequence of limb compression. Research shows explicitly that pulse oximetry exhibits greater deviations from Doppler-derived AOP in the lower limbs, indicating reduced precision when setting pressure for standardized BFRT protocols [17].

AOP/LOP-calibrated BFRT devices with individualized occlusion assessment are often regarded as the gold standard method [21,22]. The FitCuffs system enables such personalized AOP/LOP determination through manual pressure calibration, meeting the basic requirement for custom occlusion levels. However, it lacks the auto-regulated pressure features of fully automated BFRT systems, which continuously adapt to changes in limb position, muscle tension, or cuff movement. In this study, AOP/LOP was manually set at the start of the intervention and was not continuously re-adjusted during the walking bout. These limitations should be acknowledged, as any inaccuracies in the initial AOP/LOP measurement could affect the physiological stimulus delivered during the intervention.

One notable finding of this study was a significant main effect of time for PPT, indicating a slight decrease in pain tolerance from pre- to post-assessment, regardless of condition. This suggests that the walking task alone—not the application of BFR—may have briefly influenced nociceptive processing. Short bouts of low-intensity aerobic activity can temporarily alter pain sensitivity, potentially through transient mechanical loading, mild metabolic stress, or sympathetic activation before returning to baseline [23]. The absence of a condition × time interaction confirms that BFR did not affect this general pre–post response.

In contrast, neither ROM nor myofascial stiffness showed significant changes from pre- to post-assessment. This absence of time effects suggests that the short-duration, low-load walking protocol did not produce a measurable impact on passive flexibility or the viscoelastic properties of fascial and muscle tissue. These findings align with previous research indicating that meaningful modifications in passive tissue properties typically require sustained mechanical loading—such as stretching protocols or manual techniques like myofascial release—rather than brief sessions of low-intensity aerobic activity [24,25]. The lack of condition × time interactions further confirms that BFR neither enhanced nor diminished these responses compared to the control.

Importantly, the ROM results should also be interpreted considering the selected outcome measure. The SLR was chosen as a standardized and clinically relevant measure of posterior-chain flexibility and hip flexion ROM, with high measurement reliability using digital goniometry [14,15]. However, because the cuff was applied proximally on the thigh, SLR provides only an indirect proxy for local thigh-related effects. More joint-specific measures (e.g., knee ROM) may have been more sensitive to localized changes and should be included in future studies. Therefore, the selection of SLR should be considered a methodological limitation when interpreting the ROM findings.

The five-minute duration was chosen to represent a minimal, clinically feasible low-intensity walking task rather than to induce pronounced metabolic or structural adaptations. Although blood flow restriction can amplify metabolic stress and pain-modulatory mechanisms even during low-load exercise, the magnitude of these responses depends on the applied stimulus. The magnitude of these responses depends on the applied stimulus and may follow a dose–response relationship [1,6,18]. Short-duration aerobic bouts may therefore be insufficient to induce measurable acute changes in pain sensitivity, flexibility, or tissue stiffness. Consequently, the absence of significant BFR-specific effects may partly reflect an insufficient exercise “dose”, which may have reduced statistical sensitivity to detect minor acute effects.

A relative pressure of 70% AOP was selected because it falls within the commonly recommended range for blood flow restriction (40–80% AOP), as outlined in recent consensus statements and methodological papers [26,27]. Several prior studies using walking or other low-load aerobic BFR methods have also employed pressures in this upper part of the range [1]. Additionally, because of the non-automated cuff system and the inability to adjust pressure during movement, a slightly higher initial pressure was chosen to ensure that adequate pressure remained within the target range during exercise. The dynamic nature of walking may introduce slight variations in the effective occlusion stimulus. Like other practical BFR-walking protocols, cuff pressure was set individually. However, minor fluctuations in effective pressure during gait cannot be entirely excluded due to changes in limb circumference and movement-related cuff settling. Importantly, this reflects a typical applied BFR setting and supports the external validity of the protocol, but it may contribute to inter-individual variability in acute responses.

From a clinical perspective, external validity is limited by the participants’ young age. Older adults or individuals with musculoskeletal impairments often tolerate high-pressure BFRT less effectively due to discomfort from rigid cuffs and partial arterial occlusion. In contrast, some other BFRT methods use lower, cyclic, and sub-occlusive pressures that never reach complete arterial occlusion [28]. For example, one method employs low, cyclic, sub-occlusive pressures and is not designed to fully occlude the artery; therefore, it does not require AOP measurement. This difference is significant because such alternative BFRT methods have been systematically used among older adults.

In contrast, high-pressure BFRT is less common in this group due to discomfort and safety concerns. Consequently, the current findings mainly apply to healthy young adults rather than clinical or older populations.

The present findings should be interpreted considering the study sample and statistical sensitivity. Participants were healthy young adults with high baseline tissue quality and homeostatic capacity, which may reduce responsiveness to brief low-intensity interventions and limit the likelihood of detecting acute improvements in ROM or myofascial stiffness. Although the a priori power analysis was based on medium effect sizes, smaller effects may be more realistic for the present short-duration protocol. Therefore, the sample size may be limited in its ability to detect subtle changes, and the null findings should be interpreted accordingly. Relatedly, a limitation of this study is that participants were analyzed as a pooled sample, which may mask inter-individual variability in response to BFR. Sex-related differences in pain sensitivity and vascular responses to occlusion may contribute to heterogeneous acute effects; however, although our sample included women (n = 10) and men (n = 12), it was not powered to test subgroup interactions. In addition, baseline fitness and habitual physical activity were not formally quantified, precluding analyses of fitness-related responder patterns. Future studies should include standardized fitness measures and be adequately powered to examine sex- and fitness-related variability, as well as responder–non-responder profiles.

A key methodological consideration of the present study is the use of a contralateral within-participant design, in which both conditions were applied simultaneously during the same session. While this approach reduces between-subject variability, it also increases the likelihood of systemic effects influencing both limbs. This is particularly relevant for pain-related outcomes, as both aerobic exercise and blood flow restriction have been shown to elicit exercise-induced hypoalgesia mediated by central mechanisms [6,23]. Consequently, systemic hypoalgesic effects may have attenuated potential limb-specific differences in pressure pain thresholds, thereby reducing the sensitivity to detect BFR-specific effects. This limitation should be considered when interpreting the null findings for pain modulation.

Given its acute nature, the present study examined immediate responses to a single, brief bout of low-intensity BFR walking. While this approach is appropriate for detecting short-term neuromechanical and pain-related changes, it cannot capture cumulative or adaptive responses that may develop with repeated exposure. Therefore, the absence of measurable acute effects under the present protocol should not be interpreted as evidence against the potential therapeutic relevance of BFR walking in longer-term rehabilitation or training contexts.

Several additional methodological aspects further limit the scope of interpretation. First, pressure pain sensitivity was assessed at a single measurement site, and no systemic nociceptive assessments were included; thus, potential regional or generalized hypoalgesic responses may have been overlooked. Second, the applied stimulus was limited to one pressure level and a short intervention duration, which may not reflect the range of BFR-walking protocols used clinically and could contribute to the observed null effects. Future work should therefore systematically vary intervention duration and occlusion pressure, incorporate multiple local and remote pain assessment sites, and extend investigations to clinical and older populations, who may respond differently due to altered pain processing, tissue properties, and exercise tolerance. Finally, comparative studies evaluating the present approach against automated or continuously pressure-regulated BFR systems under standardized conditions would help clarify differences in tolerability, stimulus stability, and clinical relevance.

## 5. Conclusions

In healthy adults, a single five-minute bout of low-intensity BFR walking did not produce measurable acute changes in hip range of motion, myofascial stiffness, or pressure pain threshold beyond the general pre- and post-effects observed with walking alone. These findings suggest that, under the present short-duration protocol, BFR did not add detectable short-term neuromechanical or pain-modulatory effects. Further studies should investigate longer and/or repeated BFR-walking protocols, different occlusion pressures, and clinically relevant populations. They should compare manual and automated pressure-regulated systems to clarify dose–response relationships and potential therapeutic relevance.

## Figures and Tables

**Table 1:** Baseline Characteristics of the study sample (limb-level data).

			95% Confidence Interval		
	N	Mean	Lower	Upper	SD
Gender (n) female/male	43	12/10			
Age (years)	43	28.58	26.75	30.42	5.96
Height (cm)	43	1.77	1.73	1.80	0.11
Weight (kg)	43	70.09	66.58	73.61	11.43
BMI (kg/m^2^)	43	22.35	21.58	23.12	2.50

Values are reported per limb; one participant contributed data from only one limb due to a technical issue.

**Table 2:** Descriptive data.

					95% Confidence Interval		
		Condition	N	Mean	Lower	Upper	SD
PPT	t0	BFR	21	41.187	32.360	50.01	19.393
		CG	22	43.274	34.002	52.55	20.912
	t1	BFR	21	37.816	29.242	46.39	18.836
		CG	22	37.926	29.987	45.86	17.905
ROM	t0	BFR	21	80.381	73.062	87.70	16.079
		CG	22	77.864	70.667	85.06	16.231
ROM	t1	BFR	21	78.238	70.193	86.28	17.675
		CG	22	77.818	70.553	85.08	16.387
MF	t0	BFR	21	0.901	0.769	1.03	0.290
		CG	22	0.900	0.774	1.02	0.282
MF	t1	BFR	21	0.940	0.822	1.06	0.260
		CG	22	0.958	0.774	1.14	0.414

Descriptive outcome data at pre- and post-measurement for the BFR and control limbs (n = 21 BFR limbs and n = 22 control Figs due to unilateral missing data in one participant). PPT = Pressure Pain Threshold; ROM = Range of Motion; MF = Myofascial Stiffness; BFR = Blood Flow Restriction; CG = Control Group.

**Table 3:** Tukey-HSD of time on PPT.

Time	Condition	Time	Condition	Mean Difference	SE	df	t	*p*-Tukey
Pre	BFR	Pre	CG	−2.09	6.16	41.0	−0.339	0.986
Pre	BFR	Post	BFR	3.37	1.83	41.0	1.844	0.268
Pre	BFR	Post	CG	3.26	5.89	41.0	0.553	0.945
Pre	CG	Post	BFR	5.46	5.88	41.0	0.928	0.790
Pre	CG	Post	CG	5.35	1.79	41.0	2.995	0.023 *
Post	BFR	Post	CG	−0.11	5.60	41.0	−0.020	1.000

Tukey HSD post hoc pairwise comparisons for pressure pain threshold (PPT) across time (pre vs. post) and condition (BFR vs. control). * Significant at *p* < 0.05. BFR = Blood Flow Restriction; CG = Control group.

**Table 4:** Reported sensations and adverse effects.

Reported Sensation	BFR (n)	Control (n)
Pressure/discomfort under the cuff during intervention	3	0
Burning sensation in the working musculature (exercise-related)	2	0
Burning pain during post-intervention PPT assessment	2	0
Temporary skin erythema after cuff release	1	0
Tingling sensation upon cuff release	1	0
Increased muscle soreness in BFR limb (pre-existing)	1	0

Frequencies indicate the number of participants reporting each sensation. The same participant could report multiple sensations. BFR = Blood Flow Restriction.

## Data Availability

The datasets generated and analyzed during the current study are available from the corresponding author on reasonable request.

## Supplementary Materials

The following supporting information can be downloaded at: https://www.mdpi.com/article/10.3390/jcm15031052/s1, CONSORT diagram.
